# Supplementary material for: Improving the robustness and efficiency of cell sheet protocol for breast cancer induction in animal model: A Geltrex plus Gelatin approach
Source: PLoS One. 2025 Jun 23;20(6):e0326456. doi: 10.1371/journal.pone.0326456 (PMC12184892; doi:10.1371/journal.pone.0326456)
Supplement: Attachment — Submitted filename: Response to Reviewer plos one.docx [file pone.0326456.s002.docx]

Thank you for your thoughtful feedback on our manuscript, "Improving the Robustness and Efficiency of Cell Sheet Protocol for Breast Cancer Induction in Animal Model: A Geltrex™ Plus Gelatin Approach."

**Response to Reviewer #1**

**1. Justification for studying breast cancer and the clinical relevance**

- *Comment:* The introduction should provide a more compelling justification for the study, particularly emphasizing the clinical relevance and impact. Why breast cancer?

*Response:*

We appreciate this important point. We have revised the Introduction (Paragraph 1; 5) to emphasize the high global incidence and mortality rates of breast cancer, highlighting the urgent need for in vitro models that better mimic the tumor microenvironment. The MDA-MB-231 line was selected as a representative of triple-negative breast cancer (TNBC), a highly aggressive and difficult-to-treat subtype lacking targeted therapies. Modeling TNBC in a more physiologically relevant system is crucial for improving therapeutic strategies.

**2. Justification for using metastatic cells with poor junctions and trying to enhance their structure**

- *Comment:* MDA-MB-231 are metastatic and lack junctional complexes. What is the rationale for using scaffolds to enhance structure in such cells?

*Response:*

This is an excellent observation. We clarified in the revised Introduction (Paragraph 7) that our aim was not to revert the metastatic phenotype but to provide a supportive scaffold that enables reproducible handling of these otherwise fragile cell sheets. Enhancing structural integrity allows better experimental manipulation (e.g., transplantation, in vivo modeling) without altering the inherent metastatic nature of the cells.

**3. Lack of robust biological rationale and weak flow in the Introduction**

- *Comment:* The biological rationale needs strengthening, and transitions between paragraphs should be improved.

*Response:*

The introduction was rewritten.

**4. Figure-related comments**

**Figure 1**

- *Comment:* Add quantitative comparisons between cell sheets.

*Response:*

We appreciate your request for quantitative analysis of the fabricated cell sheets. Unfortunately, upon detachment, the MDA-MB-231 cell sheet did not retain its integrity, leading to significant damage. This condition limits our ability to conduct meaningful quantitative assessments. Consequently, due to the extent of the damage, performing quantitative analysis may not provide valuable data. However, we have previously investigated HUVEC and other intact cell sheets. For reference, please see Alshareeda, A.T., Sakaguchi, K., Abumaree, M., Mohd Zin, N.K., and Shimizu, T. (2017). *The potential of cell sheet technique on the development of hepatocellular carcinoma in rat models*. PLoS One, 12(8), p.e0184004.

- *Comment:* Test additional cell types and Fragility of MDA-MB-231 sheets needs optimization.

*Response:*

- Testing Additional Cell Types: We appreciate your suggestion regarding the exploration of additional cell types. However, our current study specifically focuses on the MDA-MB-231 cell line, which is known for its fragility. The improvements we present in this paper are specifically tailored to enhance the robustness of the MDA-MB-231 cell sheet protocol. We have previously published findings with other cell types, demonstrating our broader research scope. For reference, please see Alshareeda, A.T., Sakaguchi, K., Abumaree, M., Mohd Zin, N.K. and Shimizu, T., 2017. The potential of cell sheet technique on the development of hepatocellular carcinoma in rat models. PLoS One, 12(8), p.e0184004.
- Optimization of MDA-MB-231 Sheets: We acknowledge the observed fragility of the MDA-MB-231 cell sheet. As discussed, the optimizations we implemented successfully resulted in a stable cell sheet, which is the primary focus of our work. We believe that the improvements outlined in this manuscript are sufficient for demonstrating the efficacy of our approach without the need for further studies at this time. The modifications presented in our study demonstrate a significant advancement in the robustness of the MDA-MB-231 cell sheets, as evidenced by the successful *in vivo* implantation. However, we recognize the potential for future studies to optimize the MDA-MB-231 cell sheet's integrity and will consider this in our ongoing research.

**Figure 2**

- *Comment:* Discuss batch variability and cost of Geltrex™ and Validate mechanical integrity

*Response:*

- Batch Variability and Cost Constraints: We appreciate your point regarding the variability of Geltrex™ and its associated costs. We acknowledged these factors in the revised manuscript, highlighting that while Geltrex™ enhances cell adhesion, its batch variability can impact reproducibility. Now, we also discussed potential cost implications in the revised manuscript, which are important for scalability in research settings.
- Mechanical Integrity of the Cell Sheet: We understand the need for further validation of the mechanical integrity post-detachment. However, we would like to emphasize that our *in vivo* studies demonstrated successful implantation of the cell sheets without disruption, leading to tumor development. This outcome provides evidence of the integrity and handling capacity of the sheets. We believe these results validate our approach, but we will clarify this in the revised manuscript, emphasizing our handling trials and the successful application in an animal model.

**Figure 3**

- *Comment:* Crystal violet does not distinguish live from dead cells. Inconsistent staining may reflect cell loss or uneven adhesion.

*Response:*

- Viability Uncertainty: We appreciate your point about crystal violet staining primarily indicating DNA presence rather than distinguishing between live and dead cells. In our study, we utilized crystal violet to assess overall cell density and adhesion in the cell sheets. While it does not provide direct viability data, it serves as a useful qualitative indicator of cell presence and general sheet integrity. This is now clarified in the revised manuscript, emphasizing that further viability assays could complement our findings for future studies.
- Inconsistent Staining: We acknowledge your observation regarding inconsistent staining, which may suggest cell loss or uneven adhesion. Our results indicate that while the protocol improves sheet formation, optimization is indeed necessary for achieving uniform adhesion. We will address this in the manuscript, highlighting the need for further refinement in future research. However, we believe the modifications presented in our study demonstrate a significant advancement in the robustness of the MDA-MB-231 cell sheets, as evidenced by the successful *in vivo* implantation.

**Figure 4**

- *Comment:* Tumor size alone is not enough: The similarity in tumor size does not confirm functional similarity. The cell sheet model may produce different histopathological features compared to orthotopic injection.
  Were all mice able to form tumors in both conditions?
  Further analysis is needed to assess tumor histology, vascularization, and invasion potential.

*Response:* Thank you for your valuable feedback regarding our study.

- The main goal of this research was to improve the integrity of the cell sheet, and we have successfully achieved this improvement. By demonstrating the ability of the modified cell sheets to induce tumor formation *in vivo*, we confirm that the cells are viable and functional.
- Tumor Size and Functional Similarity: While we recognize that tumor size alone does not indicate functional similarity, our primary objective was to ensure the viability and integrity of the cell sheets. The successful tumor development demonstrates that our modified protocol effectively maintains cell functionality.
- Tumor Formation in Mice: We can confirm that all mice in both experimental conditions were able to develop tumors, which supports the effectiveness of our cell sheet model.
- Histological Analysis: We conducted histological evaluations, including H&E staining, as shown in Figures 5 and 6. These analyses provide insights into tumor characteristics, and we acknowledge the importance of further exploration into histopathological features, vascularization, and invasion potential in future studies.
- We appreciate your suggestions for additional analyses, which can be valuable for subsequent research. Our current focus remains on demonstrating the integrity and viability of the cell sheets, which we believe is a significant advancement in this field.

**5. Discussion is underdeveloped**

- *Comment:* The discussion lacks depth and biological mechanisms.

*Response:*

The Discussion has been expanded interpret our findings in the context of tumor microenvironment modeling, cellular mechanics, and future applications in tissue engineering and personalized oncology. Structural support is highlighted via how scaffolds can improve cell handling without reversing phenotypic characteristics. We also speculate on how this platform might be used for drug testing and understanding metastatic progression in future work. All the comments raised by the reviewer were considered by rewriting the discussion and conclusion sections.

**6. Lack of broader cancer biology context and translational impact**

- *Comment:* No discussion of molecular pathology, targeted therapies, or cancer biomarkers.

*Response:*

We have added context on the importance of MDA-MB-231 as a model for TNBC, emphasizing the lack of biomarkers and treatment options. We now discuss how this engineered sheet model could be adapted for studying response to therapeutics, particularly those targeting the extracellular matrix or metastatic signaling pathways.

**7. Clarify manuscript type (research article vs. protocol)**

- *Comment:* The study reads more like a methodology paper than a full research article.

*Response:*

While this work does include methodological development, it also provides original findings on tumor formation, viability, and histology, warranting its classification as a research article. We have clarified this dual nature in both the Abstract and Discussion, acknowledging the methodological contribution while presenting key biological insights.

**8. Weak conclusion and superficial interpretation**

- *Comment:* Enhance the conclusion with a deeper interpretation and clinical implications.

*Response:*

The Conclusion has been rewritten to better synthesize the study’s main outcomes, link findings to future applications (e.g., patient-derived sheets, drug testing platforms), and position this model as a step toward more physiologically relevant in vitro cancer systems.

**Response to Reviewer #2**

We sincerely thank Reviewer #2 for the positive and encouraging comments. We are pleased that the structure, clarity, and novelty of the manuscript were well received.

Finally, we have incorporated several improvements suggested by Reviewer #1, including expanded discussion to further strengthen the scientific depth and translational relevance of our findings.

Thank you
