# Supplementary material for: Improving the robustness and efficiency of cell sheet protocol for breast cancer induction in animal model: A Geltrex plus Gelatin approach
Source: PLoS One. 2025 Jun 23;20(6):e0326456. doi: 10.1371/journal.pone.0326456 (PMC12184892; doi:10.1371/journal.pone.0326456)
Supplement: Attachment — Submitted filename: Response to Reviewer plos one round 2.docx [file pone.0326456.s003.docx]

We sincerely thank the reviewer for the thoughtful and detailed evaluation of our revised manuscript. We greatly appreciate the recognition of the improvements made thus far and the constructive suggestions to further enhance the scientific rigor and translational relevance of our work. Below, we address each point raised:

**1. Quantitative Analysis of Cell Sheet Integrity**

Comment: The manuscript lacks a quantitative comparison between native and modified cell sheets in terms of structural integrity.

Response:

At the stage of the research, the primary aim of this study was to provide a proof-of-concept for this approach. We appreciate the reviewer's suggestion to include a quantitative comparison. However, we would like to clarify that the MDA-MB-231 cell line used in our study known to have weak or absent cell junctions, which poses a significant challenge in assessing structural integrity using conventional quantitative methods in our model.

In vitro, MDA-MB-231 cells are typically cultured as a monolayer on a flat surface, such as a Petri dish, which allows for easy assessment of cell morphology and adhesion properties. However, when transitioning to a 3D environment, such as the layered gel system used in our study, the cells' behavior and interactions with the surrounding matrix change dramatically.

Moreover, the translation of cell sheets from an in vitro setting to an in vivo environment, such as animal tissue, introduces additional complexities, including changes in oxygen tension, nutrient availability, and mechanical stress. These factors can significantly affect the structural integrity of the cell sheet, making it difficult to design a quantitative assay that accurately reflects the cell sheet's behavior in both settings.

To evaluate the integrity of the cell sheets, we employed a complimentary approach. After building up our 3D model, we re-plated the cell sheets in a normal Petri dish to assess their ability to re-attach and grow. Notably, we found that the cells re-attached normally within 24 hours and continued to grow and proliferate over subsequent days. This is evident in Figure 2.C, where we used Crystal Violet staining after fixing the whole plate to demonstrate normal cell growth and morphology.

The fact that the cell sheets were able to re-attach and grow normally in a 2D environment after being cultured in the 3D Geltrex™ plus Gelatin system suggests that the modified cell sheet protocol does not compromise the intrinsic properties of the MDA-MB-231 cells. Instead, it appears to maintain their viability and ability to grow and proliferate.

While this approach may not provide a direct quantitative measurement of structural integrity, it offers a functional assessment of the cell sheets' ability to adapt to different environments and maintain their cellular properties. We believe that this complementary approach, combined with the qualitative observations of improved cell sheet morphology and stability in the modified system, provides a comprehensive understanding of the cell sheet's behavior and integrity in the context of our study.

Nevertheless, we have now included a rationale for the absence of such quantitative analysis in the revised manuscript (Results section, page 14, lines 327 – 334).

**2. Viability Assays Beyond Crystal Violet**

Comment: Reliance on crystal violet staining limits interpretation of cell viability.

Response: As clarified in comment 1, we appreciate the reviewer's comment. While Crystal Violet staining is a widely used method to evaluate cell growth and morphology, we acknowledge that it may not provide a comprehensive understanding of cell viability.

However, we would like to clarify that our use of Crystal Violet staining in Figure 2.C was intended to demonstrate the ability of the cell sheets to re-attach and grow in a 2D environment after being cultured in the 3D Geltrex™ plus Gelatin system, rather than to provide a definitive assessment of cell viability. The fact that the cells were able to re-attach and grow normally, as evidenced by the Crystal Violet staining, suggests that the modified cell sheet protocol does not compromise the intrinsic properties of the MDA-MB-231 cells.

Moreover, Crystal Violet staining is one of the methods to detect maintained adherence of cells, which is a critical aspect of cell sheet integrity. By using this method, we were able to assess the ability of the cell sheets to maintain their adherence properties after being cultured in the 3D system, and the results suggest that the modified protocol supports the maintenance of cell adherence. This is an important consideration, as cell adherence is a key factor in determining the overall integrity and functionality of the cell sheets.

3. Molecular and Translational Relevance to TNBC

Comment: The discussion currently lacks depth on triple-negative breast cancer (TNBC)-specific molecular features or implications.

Response: We have expanded the Discussion (page 22, lines 487 – 495) to include a more detailed overview of key TNBC-specific biomarkers, including EGFR, androgen receptor (AR), and PD-L1. At the same time we want to keep the focus on the main idea which is how our methodology could be leveraged in future targeted therapies or personalized treatment strategies for TNBC patients.

4. Clarification of Manuscript Type

Comment: The manuscript’s classification (original research, methodological paper, or hybrid) is ambiguous.

Response: Thank you for pointing this out. We have now clearly stated in both the Abstract and Introduction that this work should be considered “original research” presenting original research findings alongside methodological advancements. We have also ensured alignment with the journal’s submission categories (page 3, lines 57-58).

5. Discussion of Scaffold Material Alternatives

Comment: No alternative scaffold materials discussed despite acknowledged cost and variability concerns.

Response: We acknowledge the reviewer’s valuable point. In the revised Discussion (page 20-21, lines 454 – 457), we have briefly mentioned alternative scaffold options, including scalable synthetic polymers (e.g., polyethylene glycol [PEG]-based hydrogels) and decellularized matrices, which could be alternatives for future optimization and clinical translation.

We are grateful for the reviewer’s insightful feedback, which has helped us significantly strengthen the manuscript. We have carefully incorporated all suggestions and believe the revised version better addresses concerns regarding scientific rigor, reproducibility, and clinical relevance.
